# Small-animal 360-deg fluorescence diffuse optical tomography using structural prior information from ultrasound imaging

**DOI:** 10.1117/1.JBO.25.3.036001

**Published:** 2020-03-03

**Authors:** Pei-An Lo, Shih-Po Su, Huihua Kenny Chiang

**Affiliations:** National Yang-Ming University, Institute of Biomedical Engineering, Taipei, Taiwan

**Keywords:** fluorescence tomography, fluorescence diffuse optical tomography, diffuse optical tomography, dual-modality imaging, image reconstruction

## Abstract

We demonstrate dual modality of free-space fluorescence diffuse optical tomography (FDOT) and handheld ultrasound (US) imaging to reveal both functional and structural information in small animals. FDOT is a noninvasive method for examining the fluorophore inside an object from the light distribution of the surface. In FDOT, a 660-nm continuous wave diode laser was used as an excitation source and an electron-multiplying charge-coupled device (EMCCD) was used for fluorescence data acquisition. Both the laser and EMCCD were mounted on a 360-deg rotation gantry for the transmission optical data collection. The structural information is obtained from a 6- to 17-MHz handheld US linear transducer by single-side access and conducts in the reconstruction as soft priors. The rotation ranges from 0 deg to 360 deg; different rotation degrees, object positions, and parameters were determined for comparison. Both phantom and tissue phantom results demonstrate that fluorophore distribution can be recovered accurately and quantitatively using this imaging system. Finally, an animal study confirms that the system can extract a dual-modality image, validating its feasibility for further *in vivo* experiments. In all experiments, the error and standard deviation decrease as the rotation degree is increased and the error was reduced to 10% when the rotation degree was increased over 135 deg.

## 1 Introduction

Recently, fluorescent molecular imaging has been widely used for verifying drug efficacy, and tumor and metabolism monitoring in preclinical diagnosis.^1^^–^^4^ Various fluorescent imaging platforms have been developed for noninvasive *in vivo* measurement of functional imaging.^5^^–^^10^ Fluorescence diffuse optical tomography (FDOT) is one of these imaging techniques that has the following advantages: suitable penetration depth, reasonable cost, technological readiness, and convenience.

Accordingly, researchers have developed hybrid imaging systems by combining anatomical and FDOT imaging systems.^11^^–^^15^ Magnetic resonance imaging (MRI) provides high-quality structural information about soft tissues.^16^^–^^18^ Davis et al.^17^ used MRI information to reconstruct small-animal-brain FDOT images. The optical information of objects is acquired by coupling through optical fibers in MRI systems. The long operating time and prohibitive price of the MRI system have limited its application and potential development in the small-animal-imaging market. On the other hand, some researchers have adopted small-animal computed tomography (CT) imaging to extract prior information for FDOT reconstruction in small-animal tumor models.^4^^,^^7^^,^^19^^–^^21^ Ale et al.^22^ used a free-space projection view of charge-coupled device (CCD)-based fluorescence data and structural information from CT to reconstruct FDOT for both simulation and a lung inflammation animal study.

The use of ultrasound (US) to acquire structural information has several beneficial features. (1) US imaging is free from radiation, which is safer for both operators and the imaged objects. (2) With smaller size and lighter weight than CT or MRI, the US transducer can be mounted on any machine or handheld. (3) The US transducer is easy to use. (4) The US imaging provides real-time, good soft tissue contrast without requiring contrast agents. (5) It is cheaper than both CT and MRI.^23^ (6) Over the past few decades, robotic US systems have been used in clinical use, and several semiautomatic or fully automatic US imaging systems have been presented for tumor segmentation and contour extraction.

Several studies have adopted the US system to assist in optical imaging and to provide prior information to constrain tomography reconstruction.^24^^–^^29^ Zhu et al.^28^^,^^29^ developed a hybrid “optical + US” probe to distinguish between benign and malignant breast lesions. Optical fibers were used to collect the reflected and diffused light in the tumor region to reconstruct a diffuse optical tomography (DOT) image. Li et al. used the structural information from a US image acquired by a single-element transducer to reconstruct FDOT images. Both the single-element transducer and the optical fiber were mounted on a translation stage and placed under a water bath to scan the object. The bottom of the object needed to be exposed in the water at a temperature of 37°C. The results of *in vivo* experiments indicated that the dual-modality imaging system could extract both functional and anatomical information.^25^^–^^27^ Flynn et al. used a US transducer directly to produce a US image and mounted it with optical fibers that were in reflection. The high-frequency US-guided FDOT allowed the skin layers to be measured quantitatively.^24^

Within the past five years, US-guided DOT has been used to reconstruct the hemoglobin concentrations of lesions and assist for cancer diagnosis, monitoring, and treatment.^30^^,^^31^ Althobaiti et al. evaluate the performance of the direct regularization imaging technique using the US priors in the reconstruction of DOT. They conduct both phantom and clinical experiments. The results show an improvement in the characterization of malignant and benign breast lesions.^31^ For the FDOT, different algorithms have been presented for improving the quality of FDOT.^32^^–^^35^ Chong et al.^33^ presented an algorithm to overcome the singular value decomposition-based limitation in frequency domain FDOT. Ducros et al. demonstrated an FDOT reconstruction algorithm for highly heterogeneous samples. They reconstructed the optical inhomogeneity map using continuous wave measurements and incorporated it in the reconstruction of FDOT to improve the quality.^34^ In our previous study, we used limited structural information to reconstruct the FDOT using the adaptive prior information method.^35^

In this study, we developed an innovative 360-deg-rotation transillumination FDOT and US hybrid system for small-animal imaging. The object is placed on an animal holder in the air to eliminate the inconvenience of operating in water. A US linear array transducer is used instead of a single-element transducer and profilometer. This transducer provides structural information and reveals the contour of the object. We successfully use the hybrid imaging system on the phantom, biological tissue phantom, and small animals to validate the feasibility of using medical US before achieving an economical and user-friendly solution for FDOT small-animal imaging.

## 2 Method

### 2.1 System Design

**Fig. 1:**
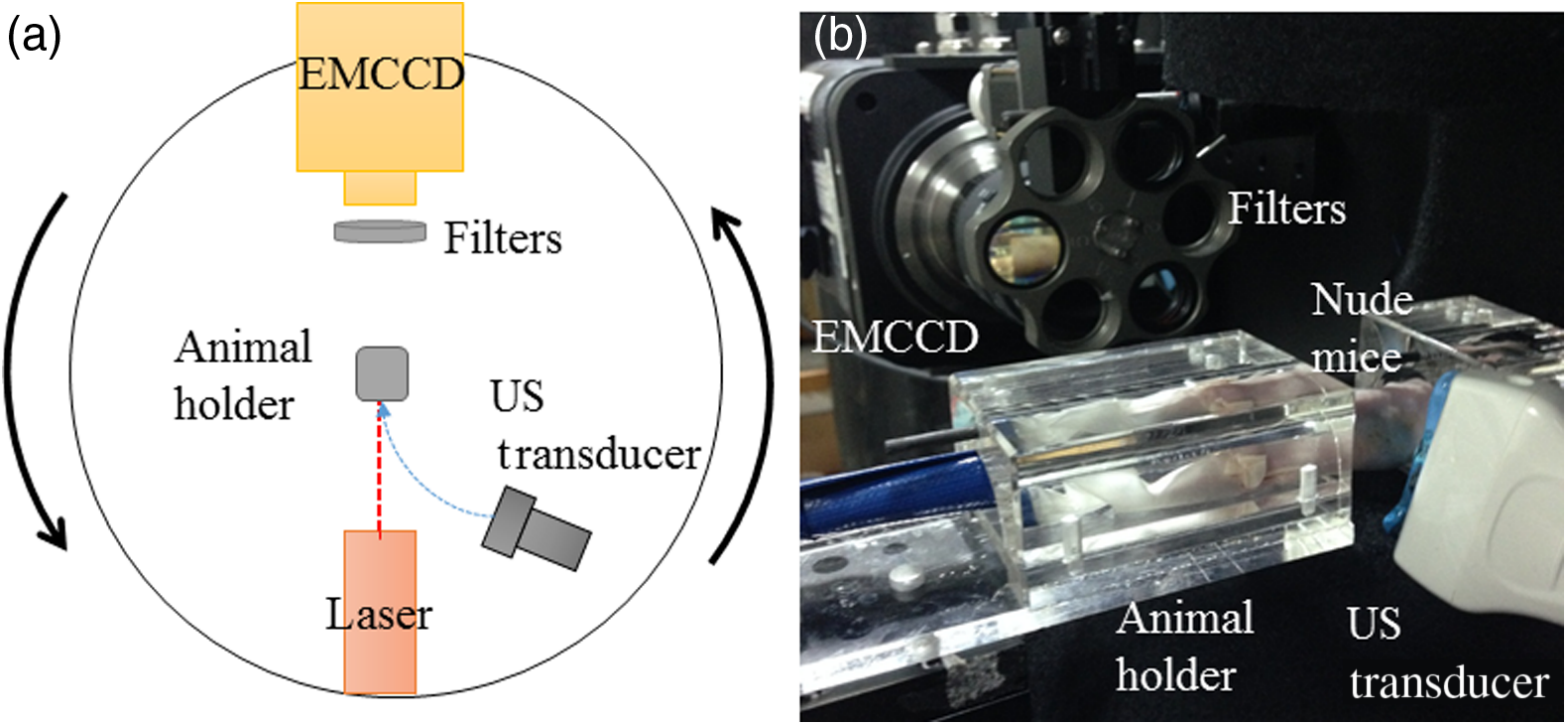
FDOT gantry. (a) Schematic of the dual-modality imaging system. (b) Animal holder, which is located in the center of the gantry.

A schematic of the system is shown in Fig. 1(a). The hybrid imaging system was realized by mounting a laser source and an electron-multiplying CCD (EMCCD; ProEM 512B-eXcelon 3-EMCCD, Princeton Instruments) on a 360-deg rotation gantry. The center of the system was a transparent acrylic animal holder. As shown in Fig. 1(b), the holder had two parts to fix the small animal while the target (the middle body) was exposed for the measurements.

A-660 nm, 40-mW diode laser (CUBE Laser, Coherent) was used as the excitation source. The emitted photons were collected by the EMCCD camera, which was coupled with a 60-mm lens (Nikon AF-S Micro) mounted on a rotation gantry to acquire a projection dataset. In front of the camera, there were two band filters for selecting the excitation and emission wavelengths.

The structural prior information and profile of the object were obtained using a handheld US transducer (Philips IU-22, L17-5, 6 to 17 MHz). After marking the US scanning position, two steps of the FDOT imaging procedure were conducted in each experiment at the same position. First, imaging was performed under white light (indoor light). In these 16 steps, white-light images were acquired to extract the object’s boundary information (left- and right-side edges). The edge location was used to coregister among the white-light image, fluorescence surface image, and US two-dimensional (2-D) tomography image in FDOT reconstruction. Then, a 660-nm laser source was used to acquire a serial fluorescence image. Images were taken every 22.5 deg over a 360-deg range in 16 different projections. For each position, the exposure time was 500 ms, and the pixel resolution was 512×512.

### 2.2 Ultrasound Imaging

The object was placed on the holder with the region of interest exposed in air for both optical and US imaging. A thin layer of the US gel was applied to the bottom of the object to examine the US image. The 2-D US tomographic information, including the tumor region and surface contour, was used in the mesh generation for the finite-element method (FEM) to calculate the FDOT. In this paper, we used a single view and obtained almost 90% of the contour and then extracted the contour manually as close to the actual structure as possible and segmented into two regions, inclusion (tumor region), and background, and used as a soft prior to constrain the FDOT reconstruction.

### 2.3 DOT Reconstruction

We used the diffuse optical equation under continuous wave to calculate the light field of excitation and fluorescence, as follows:^2^

$$\nabla \cdot (D_{x}\nabla \Phi_{x})-\mu_{ax}\Phi_{x}=S_{x},$$ (1)

$$\nabla \cdot (D_{m}\nabla \Phi_{m})-\mu_{am}\Phi_{m}=-\Phi_{x}\eta \mu_{af},$$ (2)

where Φ is the photon density, S is the isotropic source term, $\mu_{a}$ is the absorption coefficient, and D is the diffusion coefficient. The subscripts x and m represent the parameters of the excitation and fluorescence wavelengths, respectively. The fluorescence parameters are the quantum efficiency of the fluorophore η and its absorption coefficient $\mu_{af}$, which is related to its concentration.

Equation (1) is used to calculate the theoretical light field at the excitation wavelength and Eq. (2) is used to calculate the fluorescence field emitted from the excited fluorophore inside the tissue. The theoretical diffuse light field is calculated through FEM, which segments the object into many elements.^36^

To reconstruct the fluorophore concentration and the location by fluorescence acquisition from the surface of the small animal, the least squares minimization of the difference between the theoretical and experimental data is used. The minimization equation M is

$$M={\left\|\Phi^{M}-\Phi^{c}\right\|}^{2}+\lambda^{2}{\left\|L(\Delta x)\right\|}^{2},$$ (3)

where $\Phi^{M}$ and $\Phi^{c}$ represent the optical intensity generated from the measurement and calculation, respectively. Here, $\Phi^{c}$ is determined by the diffusion equation above^37^^,^^38^ and λ is the regularization parameter. In each case, we calculated the reconstruction using different values of λ. First, we chose a wide range of the regularization parameter, for example, from 10 to 1000, to reconstruct FDOT. Then, we used a smaller range and intervals, for example, from 10 to 50, to find the optimal parameters. L is the Laplacian structure, which is defined using US-derived priors,^37^ and Δx is the difference of $\eta \mu_{af}$ for each iteration.

### 2.4 Dual-Modality Imaging Fusion

The dual imaging in this system consists of US tomography and a fluorescence intensity projection image. The alignment of the CCD on the gantry needed to be carefully followed to determine the rotational axis in the optical data. For the coregistration of the two images, the following three steps need to be followed: (1) convert both the US and fluorescence images from pixel to mm scale. (2) From US tomography, we can obtain the object contour. Because the optical (FDOT) rotation axis is known, it is easy to determine the optical rotation center in US tomography. (3) The fluorescence data can be delivered in a coordinate centered to the rotation axis.

### 2.5 FDOT Experiment

To validate the system, first, phantom experiments were conducted. We utilized a 40-mm-diameter tissue-mimicking cylindrical phantom (HardBiomimic™ polyurethane phantom, INO, Inc., Canada) with following optical properties: $\mu_{a}=0.01\;{\mathrm{mm}}^{-1}$, and $\mu_{s}^{\prime }=1\;{\mathrm{mm}}^{-1}$. An 8-mm-diameter Eppendorf tube containing 0.05-mg/cc Alexa Fluor 660 (Invitrogen™) was used for representing a small-animal tumor.

**Fig. 2:**
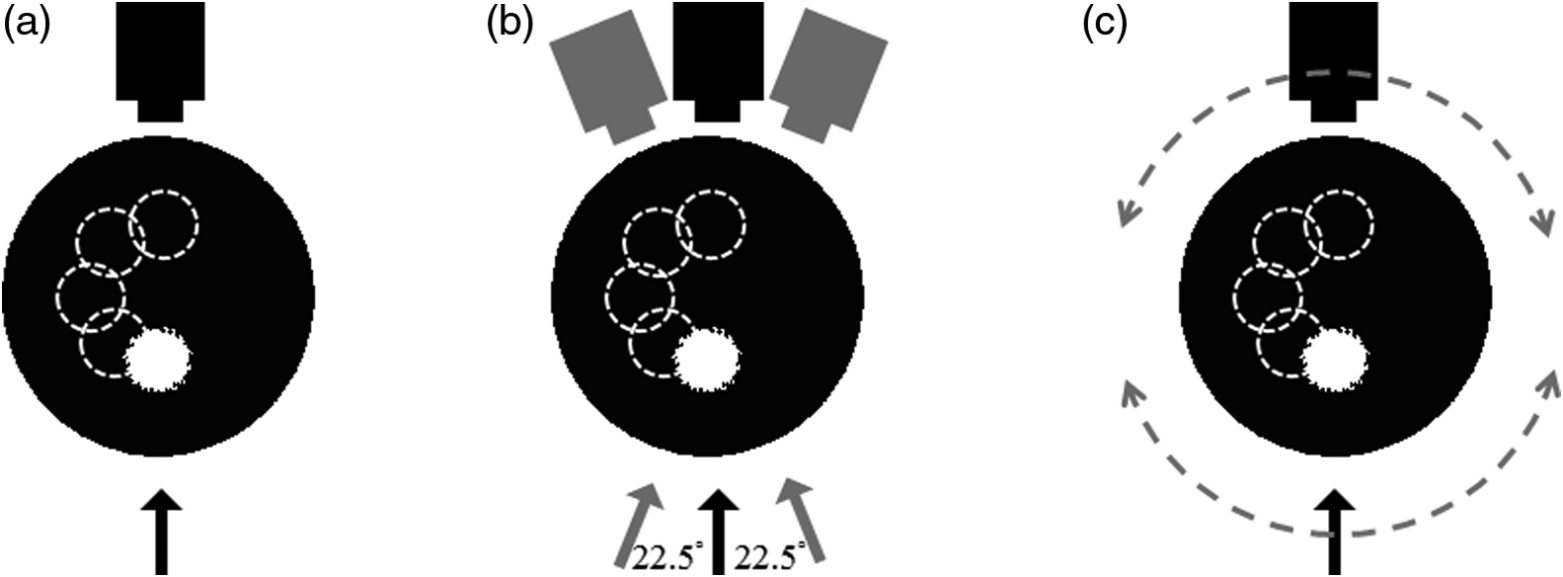
Schematic of FDOT simulation with five different sample positions. (a) Single-angle excitation, (b) with 45-deg rotation (±22.5  deg, 3 excitations), and (c) 360-deg rotation (±180  deg, 16 excitations). The arrows and dashed arrows represent the lasers and rotational direction, respectively. The dashed circles inside the phantom represent different inclusion positions.

To compare the degree of rotation, we conducted a serial simulation with different rotation degrees and sample positions. First, we compared different rotation degrees from no rotation to 360 deg. There were nine rotation cases in this simulation, corresponding to one source–detector pair [Fig. 2(a)], three source–detector pairs (45 deg, ±22.5  deg) [Fig. 2(b)], and so on, to 16 source–detector pairs (360 deg, ±180  deg). In each stage rotation case, we also conducted different sample positions, the inclusion from the bottom middle shift to the top middle, and the degree of each sample position was 45 deg.

**Fig. 3:**
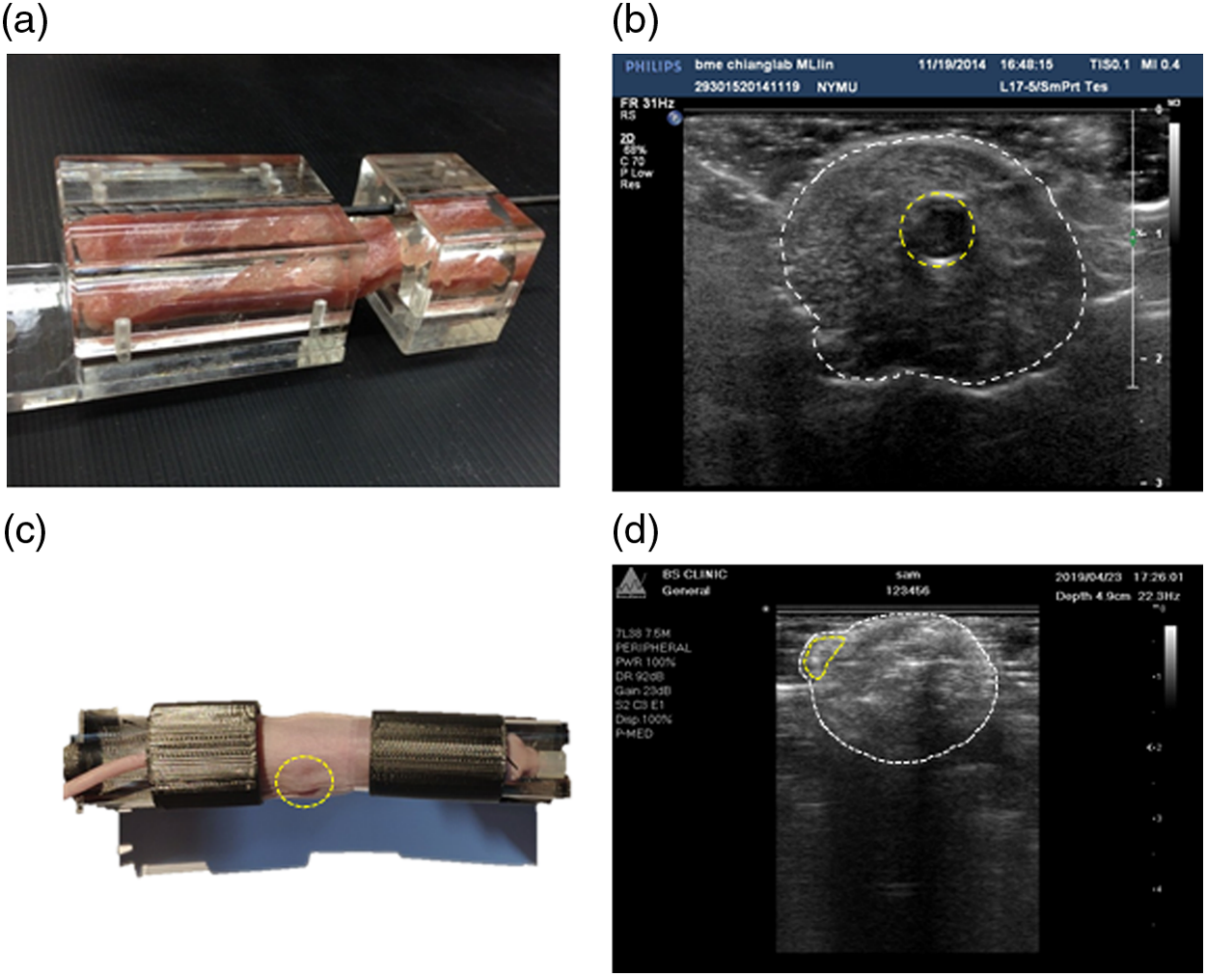
(a) Pork phantom and (b) its US image. (c) Nude mice with Matrigel fluorophore injection and (d) its US image. The white circle indicates the contour of the subject and the yellow circle indicates the inclusion (in the pork phantom) and Matrigel fluorophore injection site (in the mice).

For a nonhomogenous situation, a 23-mm×107-mm pork fillet was used to mimic a living animal tissue with a 5-mm-diameter Eppendorf tube containing 0.015-mg/cc Alexa Fluor 660, as shown in Fig. 3(a), and the US imaging was conducted as shown in Fig. 3(b). For the animal study, 5-week nude mice (BALB/c) were employed and Matrigel with Alexa Fluor 660 was implanted subcutaneously in the leg [Fig. 3(c)]. The mice received a folate-deficient diet before and during imaging and were anesthetized throughout the image acquisition. All animal studies followed the Animal Protection Act of the Laboratory Animal Center at National Yang-Ming University.

The error percentage defined as the difference of the true value and the measurement divided by the true value of the concentration. We conducted five different object positions, from the bottom middle shift to the top middle, so the mean error is the average of all errors in the five different object positions, and then we calculated the standard deviation. For the *in vivo* study, the fluorophore was injected subcutaneously into the mice, so the error here is the percentage of fluorescence outside the inclusion.

## 3 Results

### 3.1 Phantom

**Fig. 4:**
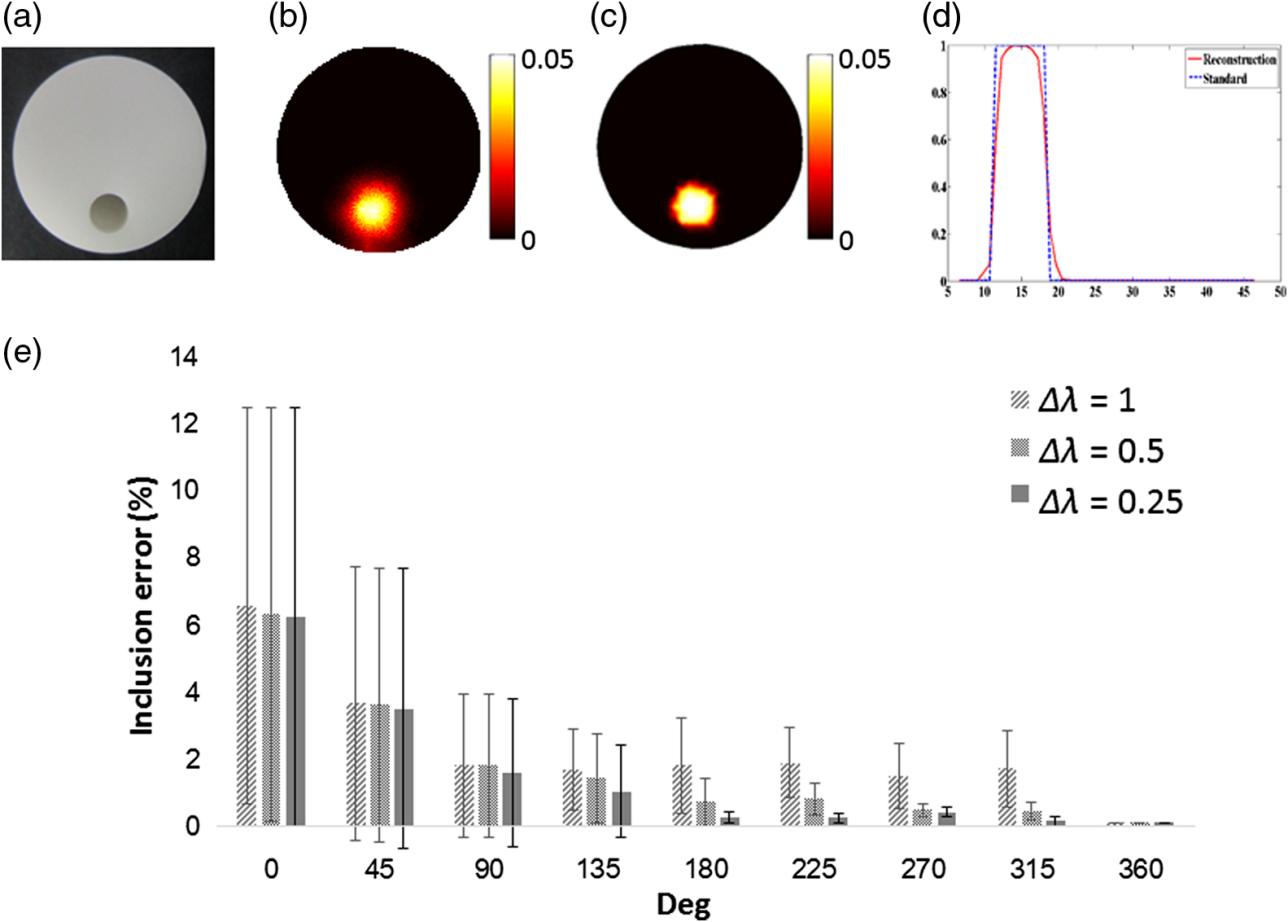
(a) Cylindrical phantom. (b) FDOT of the cylindrical phantom with fluorophore inclusion, without prior information, and (c) FDOT with US prior information. (d) Horizontal profile of the FDOT image. (e) Inclusion error for different rotation degrees (0 deg to 360 deg) and Δλs (0.25, 0.5, and 1).

An experimental cylindrical phantom is performed to validate the feasibility of the system [Fig. 4(a)]. Prior information is obtained from US imaging, where the contour of the phantom can be acquired and then used to section different areas.

The reconstruction results without prior information and with US prior information are presented in Figs. 4(b) and 4(c), respectively. The photon intensities are congruent with the position of the inserted fluorescence tube. Compared to the two reconstruction results, adding prior information improves localization and size accuracy. Figure 4(d) shows the line profile of reconstruction with the prior information, providing a more precise result. The data analysis indicates that full width at half maximum (FWHM) is 7.5 mm, which is 10% lower than that in the real situation. In the phantom study, we conduct nine rotation degrees and five object positions. In each case, we calculate the reconstruction using a different value of λ, which ranges from 1 to 10, with Δλ values of 1, 0.5, and 0.25 as shown in Fig. 4(e).

### 3.2 Pork Phantom

**Fig. 5:**
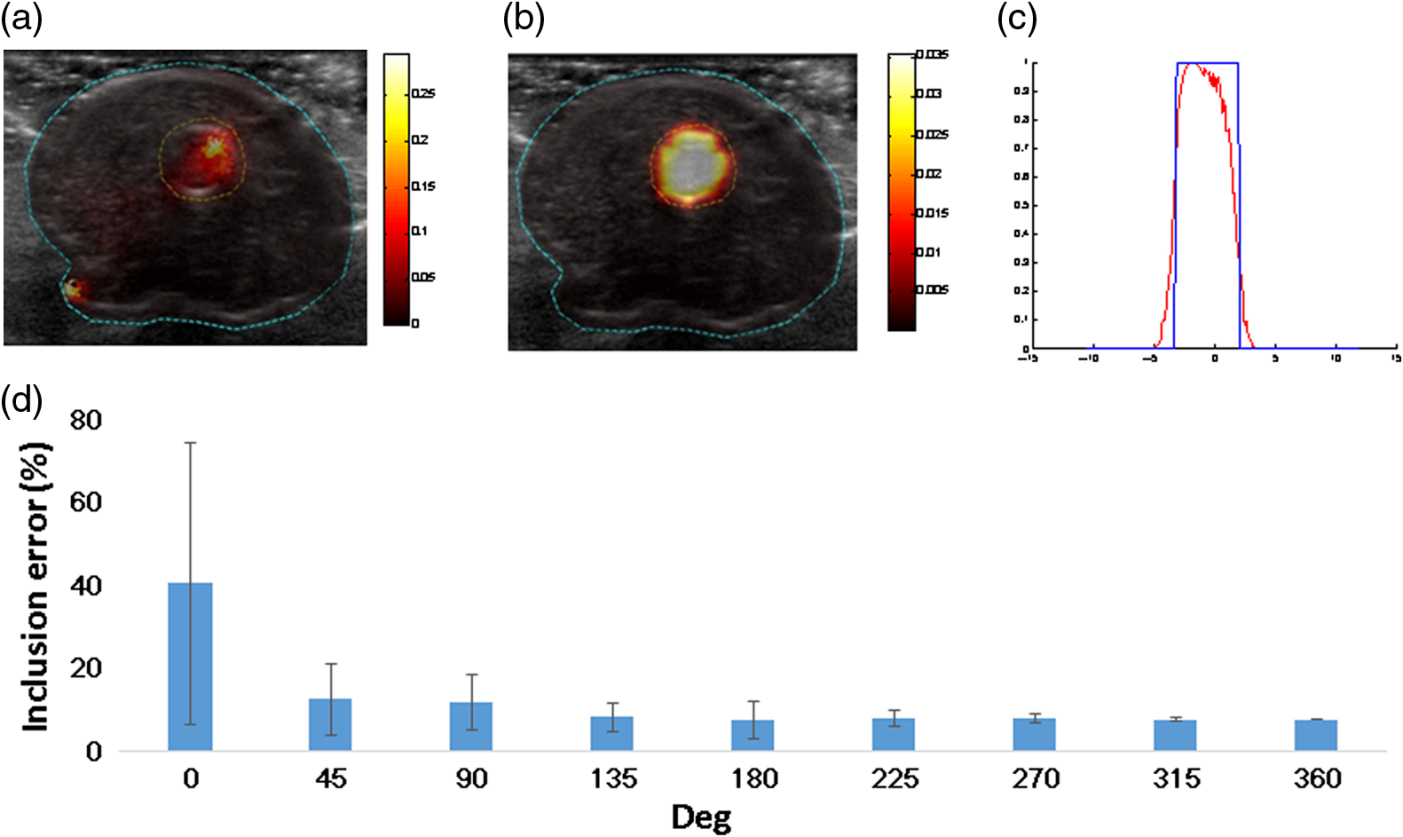
(a) FDOT of the pork phantom without prior information and (b) FDOT with US prior information. (c) Horizontal profile of the FDOT image. (d) Inclusion error for different rotation degrees (0 deg to 360 deg).

To validate the system further, we experiment with pork fillet (Fig. 5). The tissue is assigned to have $\mu_{a}=0.03\;{\mathrm{mm}}^{-1}$ and $\mu_{s}^{\prime }=1.5\;{\mathrm{mm}}^{-1}$. A 5-mm-diameter Eppendorf containing 0.015-mg/cc Alexa Fluor 660 was inserted into the pork tissue, and the $\mu_{a}$ and $\mu_{s}^{\prime }$ refer to the optical properties of the pork. The prior information and contour are acquired by US imaging.

The reconstruction results without prior information and with US prior information overlaid on the US images are presented in Figs. 5(a) and 5(b), respectively. We use λ ranging from 100 to 1500 and Δλ=100. The photon intensities are congruent to the position of the inserted fluorescence tube. Compared to the two reconstruction results, adding prior information improves localization and size accuracy, as shown in Fig. 5(c). The data analysis indicates that FWHM is 5 mm, which is 3.5% lower than the real situation. The reconstructed concentration of the fluorophore compared to the true value is 7.8% lower. Figure 5(d) provides a concentration error of inclusion from different rotation degrees, 0 deg to 360 deg. In the pork phantom, we also conducted five different object positions, from the bottom middle shift to the top middle. The average is converged when the degree exceeds 45 deg, but the standard deviation is converged until 315 deg, confirming that the use of 360 deg improves fluorescence image reconstruction.

### 3.3 *In Vivo* Results

**Fig. 6:**
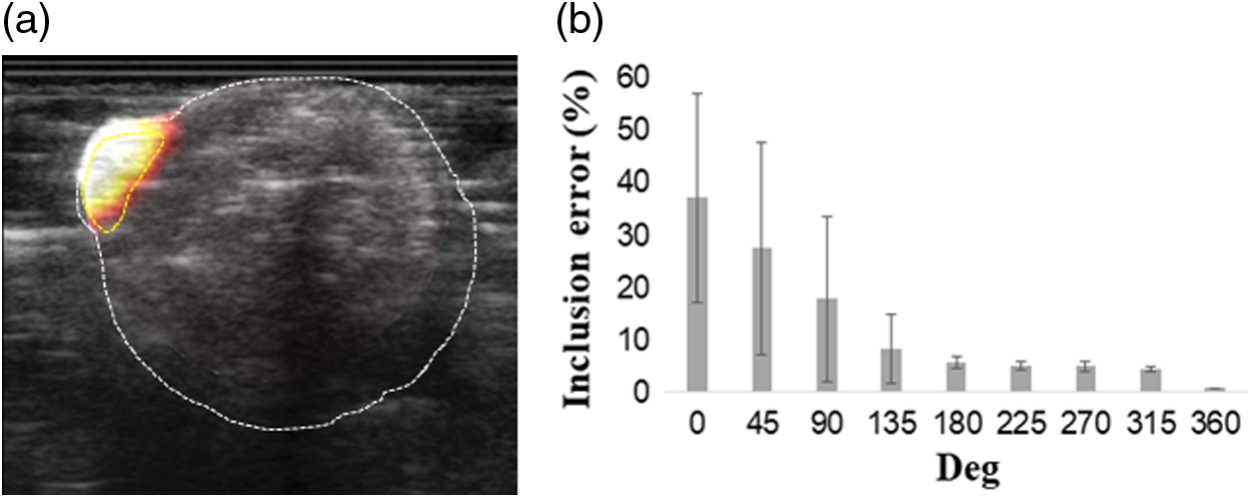
(a) FDOT of Matrigel fluorophore-injected nude mice and (b) the inclusion error for different rotation degrees (0 deg to 360 deg).

Finally, we further experiment on nude mice in our 360-deg FDOT system. The optical properties of the mice are assigned $\mu_{a}=0.01\;{\mathrm{mm}}^{-1}$ and $\mu_{s}^{\prime }=1\;{\mathrm{mm}}^{-1}$. The US prior constrains the reconstruction as a soft prior. Figure 6(a) depicts the FDOT acquired for the mice and the error at different rotation degrees [Fig. 6(b)]. The FDOT shows that the location of the fluorescence was accurately reconstructed. In the reconstruction, λ was chosen from 10 to 100 with intervals of 10. The result shows that the error decreases as the rotation degree increases. In this experiment, the fluorophore injected subcutaneously into the mice, so the error here is the percentage of fluorescence outside the inclusion. The error is <10% when the rotation degree exceeds 135 deg. At 360 deg, the error is only 1%.

## 4 Discussion

To the best of our knowledge, this is the first time that a 360-deg (transillumination) FDOT imaging system combined with a handheld medical US system has been presented. In this study, we have conducted phantom, meat experiment, and *in vivo* study, and we compared different rotation angles, different positions of the fluorophore, and different regularization parameters. This shows that 360-deg scanning is advantageous and has potential for *in vivo* study.

The 360-deg free-space fluorescence data had more symmetrically probed animal volume than planar imaging systems. From all experiments, the error and standard deviation decreased as the rotation degree increased. In most cases, the error was <10% when the rotation degree exceeded 135 deg, but the standard deviation cannot be ignored, which means that the position of the fluorophore (inclusion or tumor) can yield different results. The whole-degree optical data could eliminate the limitation of the position of the fluorophore and provide high-spatial photon sampling by complete projection measurements and thus had high-quality datasets.^34^

The handheld US imaging system has several advantages. (1) To use the US linear array to measure the structural information, the operator only needs to apply US gel on the detection surface. In other studies, the US transducer and optical fiber were placed in water and the object was partially exposed in water for measurement.^25^^–^^27^ (2) The linear array simplifies the process of obtaining anatomical information compared to the single-element transducer and reduces the scanning time. (3) The medical US probe (5 to 17 MHz) provides efficient resolution for the structural information with sufficient penetration depth for the nude mice as well as the contour information and is superior to x-ray CT when no contrast agent is used.

For FDOT reconstruction, the regularization parameter, λ, balanced the iterative optical coefficients and the data-model misfit. Different regularization parameters yielded different results. There are several ways to choose the regularization parameter. In FDOT studies, the L-curve and empirical ways were commonly used for choosing the parameters. In this study, we did not observe the L-curve in all experiments. Because we knew the true distribution of all experiments, we selected the regularization parameter that had the best result. For further experiments, if the fluorophore distribution is unknown, the methods such as the discrepancy principle, quasioptimality criterion, and generalized cross validation can be used in the reconstruction.

In this study, we used meat and phantom to verify the feasibility of the system. The inverse problem of FDOT is ill-posed because the number of measurements does not provide a unique solution and is sensitive to the heterogeneities of the object. From the phantom and meat data, the structural information provided by US can ease the ill-posed problem in FDOT reconstruction, as shown in Figs. 4 and 5. In the pork experiment, the inclusion was underestimated, which may be caused by the underestimated $\mu_{a}$ and $\mu_{s}^{\prime }$ of the pork. The signal-to-noise ratio can be improved by segmenting the US image into multiple regions and assigning more specific optical parameters, such as absorption and scattering coefficients. Overall, the system can reconstruct FDOT with structural information from the US image in homogeneous and heterogeneous phantoms.

For the *in vivo* study, the dual-modality imaging system provided a noninvasive way to examine the small animal, which enables examination of the drug metabolism. The *in vivo* experiment used in this study was a subcutaneous injection of a mixture of Alexa Fluor 660 and Matrigel in nude mice (Fig. 6). The results demonstrated that 360 deg has the smallest error and standard deviation. The dual-modality image has potential for observing the drug metabolism in the small-animal study in a noninvasive way. In the *in vivo* experiment, we only segmented the mice into two regions, and the tissue optical parameters were more complicated for the quantitative calculation, so the error was defined as the percentage of fluorescence outside the inclusion, which is different from that used in the phantom and meat experiments. The results showed that the error and standard deviation are lower when the rotation degree increases, confirming that the use of 360 deg improves fluorescence image reconstruction.

There were some results that are worth noting associated with the handheld US imaging technique. First, to reduce the reverberation caused by air between the tissue and transducer, the transducer had to contact the object firmly and this process may have caused the object deformation. Second, the frequency of the US transducer used in the system was 5 to 17 MHz with a submillimeter resolution, which can observe an individual organ in nude mice (such as heart, liver, and kidney); Fig. 3(d) shows the Matrigel in the US image. The field of view (FOV) of each US probe is different, and in this study, the FOV of the probe was around 30 mm; the object that exceeded the FOV was considered for image fusion. Third, a higher-frequency transducer is needed if a better resolution is desired, but the penetration of the US would be reduced and might not be able to penetrate all mice. Therefore, the trade-off among resolution, penetration, and FOV must be considered for each experiment. Fourth, it is worth noting that clinical US has widely adopted compound imaging method to increase image resolution, eliminate artifacts, shadows, and provide better-defined tumor boundaries,^39^ such as the PHILIPS IU22 used in our study. Finally, the speed of sound changes in different media and would generate the error in the size of the object. We have investigated the difference of the sound speed in different media. The standard value of speed of sound for soft tissue is 1540±15  m/s, which results in a maximum error of 2%. The other studies showed the maximum vertical difference between nominal and measured distance for a linear array is <1.7  mm after penetrating 5.4 cm, which indicates that the error would be <3%.^40^^–^^42^

In the future, for the *in vivo* study, the US image will need to segment into multiple regions, such as bone, liver, and intestine. In addition, the optical coefficients (absorption and scattering) will need to assign to the individual region to improve the accuracy of FDOT.

## 5 Conclusion

We demonstrated a dual-modality imaging system, a rotational CCD-based FDOT combined with US imaging. The dual-imaging system was demonstrated on phantom, tissue, and mice studies. As the rotation degree increased, the error, standard deviation, and the impact of the object position were decreased. The error was reduced to 10% when the rotation degree was increased over 135 deg. This conclusion is beneficial to future research for the small-animal FDOT.
